# “Hiding in Plain Sight”: A Retrospective Clinical and Microbiological Review of Vancomycin-Dependent Enterococci at a Tertiary Care Centre—A Case Report

**DOI:** 10.3390/microorganisms14010193

**Published:** 2026-01-15

**Authors:** Ruchika Bagga, Johan Delport, Alice Kanyua, Kumudhavalli Kavanoor Sridhar

**Affiliations:** 1 Department of Pathobiology and Laboratory Medicine, Schulich School of Medicine and Dentistry, Western University, London, ON N6A 3K7, Canada; johan.delport@lhsc.on.ca; 2 Victoria Hospital, London Health Sciences Centre, London, ON N6A 5W9, Canada; 3 Department of Laboratory Medicine and Pathology, Aga Khan University, Nairobi 00100, Kenya; alice.kanyua@aku.edu; 4 Department of Laboratory Medicine & Pathobiology, University of Toronto, Toronto, ON M5S 3K3, Canada; drkumudhammc@gmail.com

**Keywords:** *Enterococcus faecium*, vancomycin-dependent enterococci, *van A/B* gene, nosocomial infection, vancomycin, antimicrobial stewardship

## Abstract

Vancomycin-resistant *Enterococci* (VRE) are established nosocomial pathogens; however, vancomycin-dependent *Enterococci* (VDE) represent a rare and underrecognized phenomenon. These organisms paradoxically require vancomycin for growth due to mutations in cell wall precursor synthesis. Limited awareness and significant diagnostic challenges associated with VDE can lead to delayed recognition and treatment failure. We report a case of vancomycin-dependent *Enterococcus faecium* isolated from a liver transplant recipient receiving oral vancomycin prophylaxis for recurrent *Clostridioides difficile* infection. The isolate failed to grow on standard media but exhibited robust growth on vancomycin-supplemented agar, confirmed by vancomycin disc diffusion testing and PCR detection of the *vanB* gene. Additionally, we reviewed four further VDE cases identified over a two-year period in our tertiary care microbiology laboratory. All patients originated from complex care settings, had significant comorbidities, and had received prolonged glycopeptide therapy. We summarize the clinical features, diagnostic findings, and microbiological challenges encountered across this case series. This series documents the first reported Canadian case of VDE and highlights the critical need for clinical vigilance and diagnostic suspicion in high-risk patients with prior enterococcal colonization and ongoing glycopeptide exposure. Laboratory findings such as failure to grow on blood agar coupled with growth around vancomycin discs should prompt specific evaluation for VDE. Our findings reinforce the necessity for targeted antimicrobial stewardship and infection prevention strategies and underscore the remarkable evolutionary adaptability of *Enterococci* under sustained antimicrobial pressure.

## 1. Introduction

*Enterococci* are Gram-positive cocci which are a normal part of human gut flora [1]. While not regarded as highly virulent, they have emerged as significant nosocomial pathogens due to their unique ability to acquire resistance to glycopeptide antibiotics [1,2,3].

The plasticity of the enterococcal genome allows acquisition of multiple resistance genes, with mobile genetic elements constituting up to 26% of their genome [1,2,3].

Vancomycin resistance in *Enterococci* is mediated by *van* gene clusters, with *vanA* and *vanB* being the most common [1,4]. Resistance involves alteration of the peptidoglycan synthesis pathway, where the terminal D-Ala-D-Ala dipeptide is replaced by D-Ala-D-Lac, dramatically reducing vancomycin binding affinity and thereby efficacy [1,4]. Vancomycin-resistant *Enterococci* (VRE) are now widespread in healthcare settings and have prompted routine active surveillance in many institutions [1].

Vancomycin-dependent *Enterococci* (VDE) represent a further evolutionary adaptation within VRE [4,5,6,7,8,9]. These unique bacterial strains paradoxically require vancomycin for growth and survival. From a molecular perspective, VDE typically arises in *E. faecium* and *E. faecalis* strains harbouring *vanB* or *vanD* operons [4,5]. Dependence is linked to mutations in the *ddl* gene, encoding the D-Ala:D-Ala ligase, which is essential for peptidoglycan precursor synthesis. These mutations impair the organism’s ability to synthesize D-Ala-D-Ala termini in peptidoglycan precursors, rendering the bacteria incapable of cell wall synthesis in the absence of vancomycin [10]. Vancomycin, by inducing the expression of the *van* gene cluster, facilitates the production of D-Ala-D-Lac, rescuing the defect and permitting bacterial growth—albeit with reduced fitness and altered metabolic profiles [6]. This phenomenon highlights the complex interplay between antibiotic pressure and bacterial adaptation presenting new challenges in both detection and treatment of these infections [6,7,11,12]. The existence of VDE underscores the remarkable adaptability of bacteria and the ongoing need for innovative approaches in antimicrobial therapy [9,11].

VDE was first reported in 1993 in the urine of a patient on long-term vancomycin therapy [10], and subsequent cases have been described in urine, blood, stool, and other body sites [6,7,13,14]. Risk factors include prolonged vancomycin exposure, immunocompromised status, and prior VRE colonization [11,14,15].

Here we report the first Canadian case of VDE in a post-liver transplant patient, along with four additional cases identified retrospectively in our institution. We also review diagnostic challenges, treatment implications, and relevant literature.

## 2. Detailed Case Description

A 59-year-old male patient, who had undergone an orthotopic liver transplant, was admitted with pancytopenia and a disseminated varicella zoster infection in October 2022. He received his first liver transplant in October 2021 due to alcoholic end-stage liver disease; however, his recovery was complicated by acute cellular rejection and biliary stricture. During this period, he was treated with solumedrol and multiple courses of antibiotics including piperacillin-tazobactam (4.5 g every 6 h for 14 days), and meropenem (1 g every 8 h for 7 days) on multiple occasions. In March 2022, he underwent a second liver transplant, which was further complicated by an *Aspergillus* infection that required three months of voriconazole (200 mg BID), as well as episodes of Cytomegalovirus (CMV) viremia managed with valganciclovir 900 mg daily and recurrent admissions for Clostridioides difficile colitis requiring oral vancomycin prophylaxis (125 mg four times daily). He had four episodes of *Clostridioides difficile* colitis prior to his current admission.

At the time of admission, his medications included valganciclovir for CMV viremia, amoxicillin-clavulanic acid (875/125 mg BID) for a perianal abscess, oral vancomycin prophylaxis for recurrent C diff infections, and atovaquone prophylaxis.

During his current admission, surveillance swabs (nasal, axilla, rectum) collected on ESwabs (Copan, Murrieta, CA, USA) were processed in the microbiology laboratory. Swabs were plated on Brilliance VRE Agar (Thermo Fisher Scientific, Waltham, MA, USA) using the WASP Lab automated streaker, incubated at 37 °C for 36 h, and pre-screened with PhenoMATRIX™. PhenoMATRIX™ is an advanced system used to pre-assess and group culture plates, which are subsequently reviewed by trained laboratory professionals.

**Figure 1:**
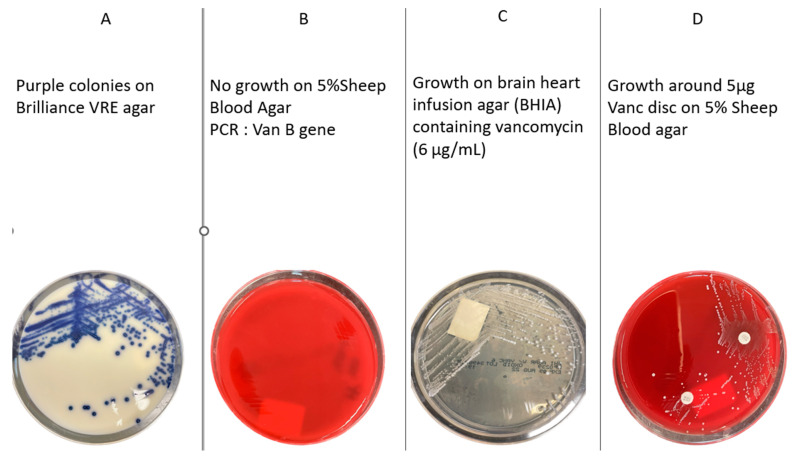
Growth characteristics of vancomycin-dependent *Enterococcus faecium*. (**A**) Brilliance VRE agar plate showing purple colonies after 36 h. (**B**) The 5% sheep blood agar showing no growth after 24 h. (**C**) Growth on BHIA containing 6 µg/mL of vancomycin. (**D**) The 5% sheep blood agar with a 5 µg vancomycin disc, showing growth restricted to the disc zone.

Colonies exhibiting a moist purple to royal blue coloration (*Enterococcus faecium*) or moist denim blue (*Enterococcus faecalis*) on Brilliance VRE agar were identified by VITEK MS (bioMérieux, Marcy-l’Étoile, France). Positive colonies were suspended in 0.5 mL saline using a Copan swab. The suspension was used to inoculate a Cepheid Xpert vanA/vanB PCR assay for detection of *vanA* or *vanB* genes. In parallel, isolates were tested on brain heart infusion agar (BHIA) containing vancomycin (6 µg/mL) with plain BHIA as a growth control (Thermo Fisher Scientific) to confirm vancomycin resistance. Concurrently, 0.5 mL of the suspension was inoculated onto 5% sheep blood agar (Thermo Fisher Scientific) and a quarter Brilliance VRE plate, incubated at 37 °C for 24 h. The isolates failed to grow on sheep blood agar but were PCR-positive for the *vanB* gene and therefore classified as vancomycin-dependent *Enterococci* (VDE) (Figure 1). These were further subcultured on 5% sheep blood agar containing a vancomycin disc (5 µg; Oxoid, Thermo Fisher Scientific, Waltham, MA, USA) to confirm the dependence phenotype. While growth dependence was stable at the time of clinical detection, the potential for reversion to VRE remains biologically plausible and is well documented in the literature.

Quality control (QC) was performed on all new batches of reagents, media, and discs at receipt and weekly thereafter; only QC-passed materials were used for clinical testing.

Whole-genome sequencing was not performed, which we acknowledge as a limitation.

A multidisciplinary team was convened to determine the most appropriate management strategy for the VDE isolate.

**Figure 2:**
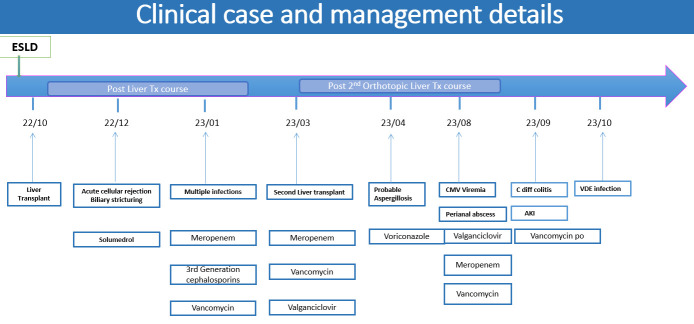
Clinical case and management details.

The primary therapeutic intervention was the discontinuation of oral vancomycin once the dependency was identified, alongside the initiation of Meropenem for the concurrent Pseudomonas aeruginosa bacteremia (Figure 2). Targeted VRE therapy (e.g., Linezolid) was withheld as the patient remained clinically stable regarding enterococcal symptoms, and the VDE was considered a colonizer.

## 3. Retrospective Case Review

A laboratory information system review identified four additional VDE isolates between 2021 and 2022 (Table 1). They were all isolated from the VRE screening swabs from rectum, axilla or groin. All were *vanB*-positive by Cepheid PCR, and all demonstrated failure to grow on plain media but growth on vancomycin-supplemented agar. Interestingly all the four cases identified retrospectively belonged to the same complex care and rehabilitation centre. Given the retrospective nature of the audit, there was limited information available on treatment plans for these patients.

**Table 1:** Review of other VDE isolated at our site between 2021 and 2022.

Patient Identifiers	Isolate	Past Medical History	History of Present Illness	Referral Centre
Patient 1/74M	Van B E *faecium*	Hypoxic resp failure, coronary artery disease, dementia	Progressive HFrEF	Complex care and rehabilitation hospital
Patient 2/94M	Van B E *faecium*	CAD, DM, ESRD, AKI	HFrEF and ESRD on dialysis, course complicated by line infections and sepsis	Complex care and rehabilitation hospital
Patient 3/64 M	Van B E *faecium*	ESRD, DM, Recurrent C diff admissions	Sepsis likely related to PD catheter infection	Complex care and rehabilitation hospital
Patient 4/47M	Van B E *faecalis*	Previously well	Ruptured aortic aneurysm, post op complicated by infections, prolonged ICU stay	Complex care and rehabilitation hospital

Abbreviations: HFrEF: Heart Failure with reduced Ejection Fraction; CAD: coronary artery disease; DM: Diabetes Mellitus; ESRD: end-stage renal disease; AKI: acute kidney injury; PD: peritoneal dialysis; ICU: intensive care unit.

We conducted a focused review of published cases of vancomycin-dependent *Enterococci* (VDE). The available literature confirms that VDE, although uncommon, represent clinically significant pathogens capable of causing healthcare-associated outbreaks. These cases most often occur in patients with prior glycopeptide exposure, established *Enterococcus* colonization, or those receiving vancomycin prophylaxis. A consolidated summary of reported cases is presented in Table 2.

Vancomycin-dependent *Enterococci* (VDE) represent a rare but clinically significant microbiological phenomenon, in which enterococcal strains acquire not only resistance to vancomycin but also a paradoxical growth requirement for it. This dual phenotype represents an advanced stage in the evolution of glycopeptide resistance and dependence and reflects the profound impact of prolonged antibiotic exposure in vulnerable patient populations. To our knowledge, this is the first reported case and case series of VDE in Canada, contributing novel insight into this underrecognized resistance mechanism.

The emergence of VDE has been predominantly documented in patients with prolonged exposure to glycopeptides, especially in those with chronic comorbidities such as end-stage renal disease (ESRD), or in immunocompromised hosts, including post-transplant patients receiving antimicrobial prophylaxis [3,4,6]. This pattern suggests that selective pressure from vancomycin, coupled with host-related risk factors (e.g., immunosuppression, indwelling catheters), plays a pivotal role in driving both resistance and dependence phenotypes.

Diagnostic recognition of VDE remains a laboratory challenge. These organisms often fail to grow on standard blood agar plates but may be recovered from culture-negative clinical samples plated onto selective media containing vancomycin—such as VRE chromogenic agar or even media intended for Neisseria or Campylobacter isolation [7]. Moreover, their growth may be observed around vancomycin discs in disc diffusion assays, a counterintuitive finding that should raise suspicion for VDE, particularly in patients with compatible clinical risk factors.

A significant limitation of this study is that whole-genome sequencing (WGS) was not performed on the identified isolates. Genomic analysis would have definitively confirmed the specific ddl gene mutations (typically D-Ala:D-Ala ligase deficiencies) and characterized the regulatory mechanisms of the *van* operon. Without WGS, we could not evaluate the presence of suppressor or compensatory mutations that facilitate the “reversion” of VDE to a traditional VRE phenotype. Furthermore, genomic data would have provided the high-resolution evidence needed to assess the clonal relatedness of the four isolates from the shared referral centre, which strongly suggests a localized outbreak or intra-institutional transmission. As several isolates were no longer viable for retrospective sequencing, our epidemiological inferences remain limited, highlighting the need for prospective genomic surveillance in future VDE clusters.

Therapeutically, discontinuation of vancomycin alone is not a reliable strategy. Several studies have demonstrated the capacity of VDE strains to revert to VRE phenotypes via compensatory or suppressor mutations, further complicating treatment [8,9]. Consequently, management often necessitates the use of alternative agents such as linezolid or daptomycin, although susceptibility testing remains essential due to potential co-resistance [16]. The role of novel agents (e.g., tedizolid, oritavancin) has yet to be evaluated in this context.

In this study, VDE was primarily identified through routine surveillance and represented colonization rather than invasive infection. This distinction is critical to avoid overestimation of the immediate clinical impact of VDE while still acknowledging its relevance in infection prevention, antimicrobial stewardship, and future infection risk. In the index patient, vancomycin exposure was ongoing and no targeted therapy was administered. Retrospective isolates were similarly detected via routine VRE screening, with limited clinical information on treatment or outcomes. As a result, the persistence, clearance after vancomycin discontinuation, or reversion to VRE within this cohort cannot be fully characterized, consistent with the heterogeneous course reported in prior studies.

From an infection prevention and control (IPAC) perspective, VDE carries a potential for nosocomial transmission comparable to conventional VRE. Stringent contact precautions, environmental decontamination, and IPAC vigilance remain essential in suspected or confirmed cases [13,15]. VDE colonization reflects significant antimicrobial selective pressure, particularly prolonged glycopeptide exposure, and may represent a transitional evolutionary state within enterococcal resistance. Although colonization alone does not require antimicrobial therapy, it has important implications for infection prevention, as colonized patients may act as reservoirs for transmission or subsequent infection under favourable conditions [13,15].

Currently, there are no evidence-based guidelines specific to VDE management. In practice, management should mirror established VRE strategies, including contact precautions, surveillance in high-risk units, and minimization of further glycopeptide exposure whenever clinically feasible.

The fact that VDE strains have been implicated in hospital-associated clusters in the United States underscores the need for surveillance and containment [15].

Antimicrobial stewardship plays a critical role in mitigating the emergence of VDE. The indiscriminate or prolonged use of glycopeptides—especially in settings where enterococcal colonization is prevalent—should be scrutinized. Judicious antimicrobial selection, guided by rapid diagnostics and resistance profiling, is essential in high-risk populations [14].

In summary, VDE exemplifies the dynamic adaptability of *Enterococci* under intense antimicrobial pressure. It represents an evolutionary endpoint wherein the organism co-opts a drug initially designed to kill it, making vancomycin a conditional growth factor. The emergence of VDE warrants a multidisciplinary response: microbiologists must remain vigilant in detecting atypical growth patterns; clinicians must balance therapeutic efficacy with resistance selection risk; and stewardship and IPAC teams must coordinate to prevent propagation. This case highlights the broader imperative to understand, detect, and manage emerging antimicrobial dependencies as an extension of resistance evolution.

## Figures and Tables

**Table 2:** Review of literature on previously reported vancomycin-dependent Enterococci (VDE).

Age	Co-Morbidities	Prior *Enterococcus* Isolation	Prior Glycopeptide Exposure	Specimen Source	Citation
32 years	DM, ESKD, post kidney transplant	Yes	Yes—vancomycin teicoplanin	Intra abdominal fluid	Tambyah PA and colleagues [6]
40 years	Type 1 DM, ESRD, kidney–pancreas transplant	Yes	Not stated	Blood culture	Tambyah PA and colleagues [6]
47 years	CML, severe GVHD, ARF, corynebacterium bacteremia	No	Yes—vancomycin	Urine	Tambyah PA and colleagues [6]
36 years	ESRD, diabetic nephropathy on HD	Yes	Yes—vancomycin	Blood culture	Stewart B and colleagues [9]
46 years	Acute cholecystitis, cholangitis and fulminant pancreatitis,	Yes	Yes—vancomycin	Urine	Fraimow HS and colleagues [10]
68 years	Lung cancer with lobectomy, CVA	Yes	Yes—vancomycin	Stool	Hwang K and colleagues [11]
36 years	Acute Myeloid Leukemia	Yes	Yes—vancomycin	Urine	Hwang K and colleagues [11]
25 years	Acute Myeloid Leukemia	Yes	Yes—vancomycin	Urine	Hwang K and colleagues [11]
60 years	Hepatocellular carcinoma with liver transplant	Yes	Yes—vancomycin	Pleural fluid	Hwang K and colleagues [11]
25 years	Multiple fracture, small bowel and urethral rupture due to traffic accident	Yes	Yes—vancomycin	Urine	Hwang K and colleagues [11]
7 months	VACTERL association	Yes	Yes—vancomycin	Urine and stool	Hwang K and colleagues [11]
27 years	ESRD secondary to reflux nephropathy, culture negative peritonitis	Yes	Yes—vancomycin	Blood culture	Majumdar A and colleagues [16]

Abbreviations: CML: chronic myelogenous leukemia; GVHD: Graft vs. Host disease; ARF: acute renal failure; HD: hemodialysis; CVA: cerebrovascular accident; VACTERL association is a group of birth defects affecting the vertebrae, anus, cardiac, trachea, esophagus, renal and limb systems.

## Data Availability

The original contributions presented in this study are included in the article. Further inquiries can be directed to the corresponding author.
